# Supplementary figures and images for: Mosquito-independent milk-associated transmission of zoonotic Wesselsbron virus in sheep
Source: PLoS Pathog. 2024 Dec 9;20(12):e1012751. doi: 10.1371/journal.ppat.1012751 (PMC11658706; doi:10.1371/journal.ppat.1012751)

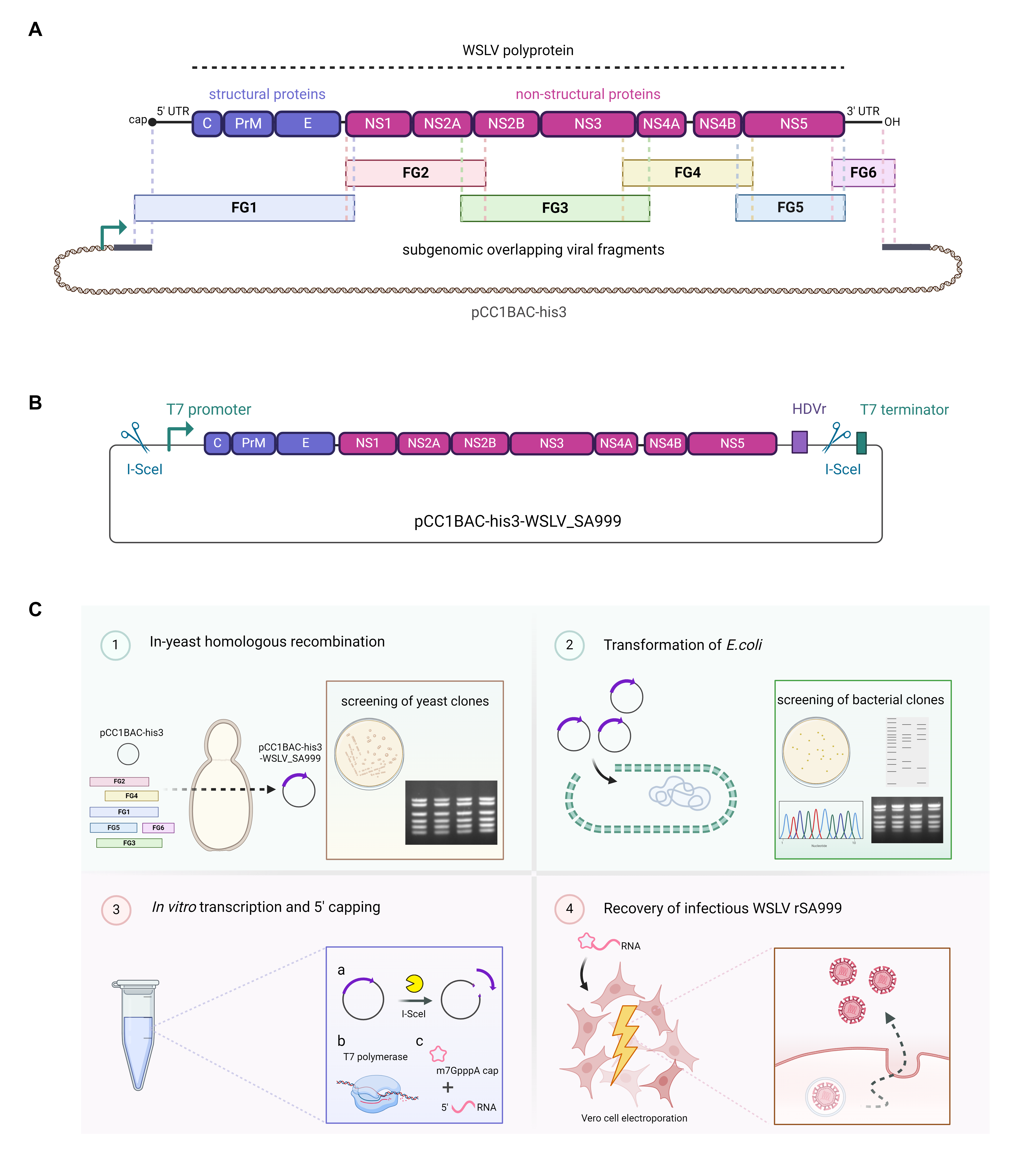

Supplement: S1 Fig — (A) Schematic representation of the 6 synthetic overlapping DNA fragments (FG1-FG6) spanning the whole genome of WSLV SA999 virus sequence and including anchors for cloning into pCC1BAC-His3 vector. (B) Map of the cloned SA999 genome. (C) Multistep process to generate infectious WSLV rSA999 virus particles (see methods for details). The figure was created using BioRender. (PNG) [file ppat.1012751.s001.png]

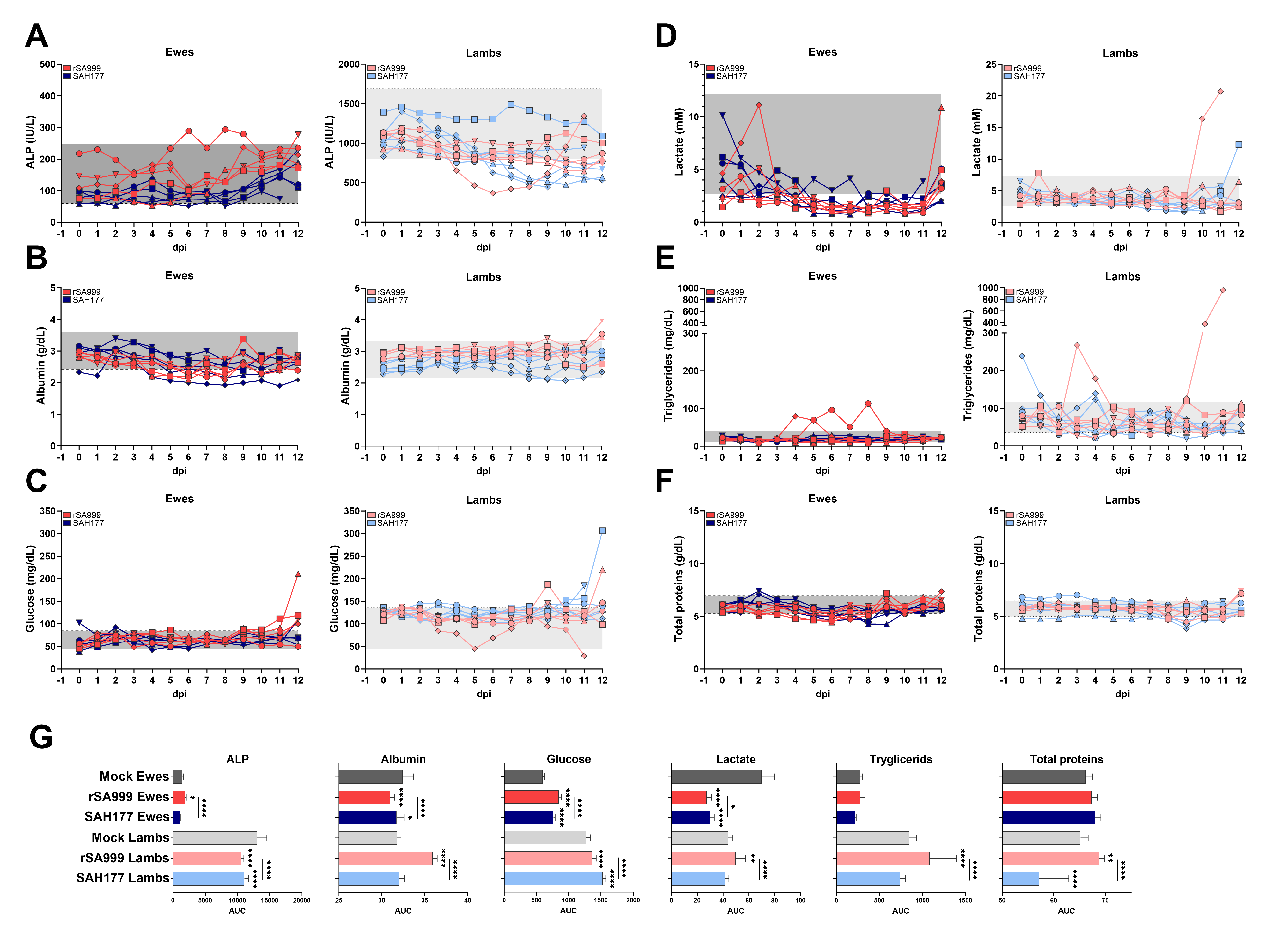

Supplement: S2 Fig — (A) Alkaline phosphatase (ALP), (B) albumin, (C) glucose, (D) lactate, (E) triglycerides, and (F) total proteins. The maximum and minimum values from the mock group of ewes and lambs were indicated for reference in gray shading background. (G) Group differences were analyzed by one-way ANOVA of the AUC with Tukey post hoc test. An asterisk on a specific group indicates differences compared to the age-matched mock; underlined asterisks indicate differences between infected groups. Statistical significance was considered for p<0.05 (***p<0.001; **** p<0.0001). (TIF) [file ppat.1012751.s002.tif]
