# Supplementary material for: Mosquito-independent milk-associated transmission of zoonotic Wesselsbron virus in sheep
Source: PLoS Pathog. 2024 Dec 9;20(12):e1012751. doi: 10.1371/journal.ppat.1012751 (PMC11658706; doi:10.1371/journal.ppat.1012751)
Supplement: S1 Appendix — (PDF) [file ppat.1012751.s004.pdf]

S1 Appendix. Sequencing Report - WSLV rSA999

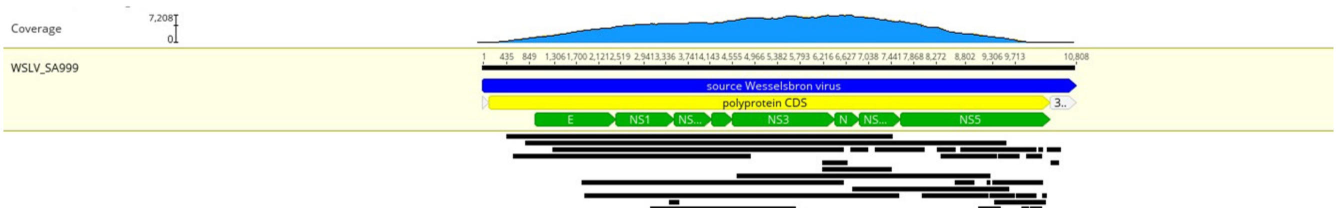

Figure 1: Figure 1 Coverage and depth graph. *39,932 fastq reads were mapped to reference sequence WSLV_SA999 (GenBank MK163943).*

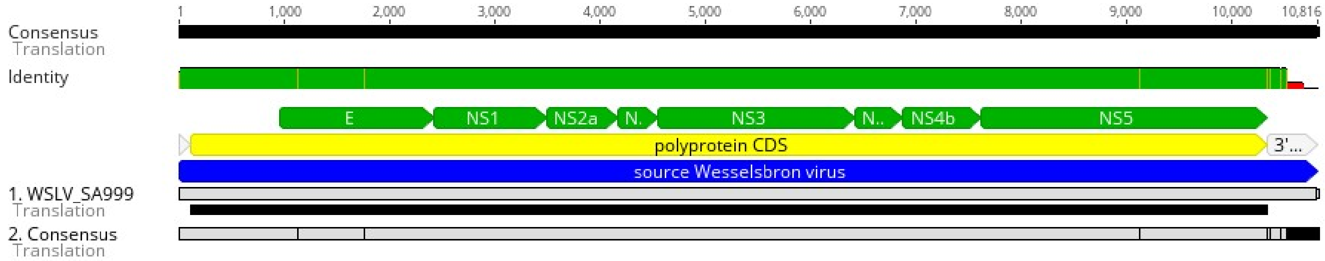

Figure 2: Alignment graph*. Alignment of the Consensus_SA999_FAX14366bc59 and WSLV_SA999. Highlighting disagreements, including ambiguous disagreements. There are only ambiguous disagmeents.*

Consensus

Threshold 65%, if there was no coverage, it called "?", if there was lower depth as 10, Geneious called "N"

Nucleotide Statistics:

Depth Mean: 4415.4 Minimum: 0   Maximum: 7208

Coverage: Ref-Seq: 98.7% (10,663 of 10,808)

5prime End: no coverage 1-2; depth below 10: 3-4

3prime End: no coverage 10674-10816; depth below 10: 10520-10673

Variant table

No variants

Geneious assembly report

✔ 18,023 of 45,444 reads were assembled to contig: WSLV_SA999 to produce SA999_FAX14366bc59 mapped to refWSLV_SA999
✖ 27,421 reads were not assembled

Assembled 89 documents using Minimap2. Show Options
Assembly Duration: 10 minutes and 21 seconds

Note: Minimap2 produced 67,353 output reads from 45,444 input reads. This may be due to Minimap2 either mapping reads to multiple locations, or splitting long reads and mapping those split reads to different regions of the reference sequence(s).

Command Line:
minimap2_linux -x map-pb --frag=yes --secondary=yes -N 5 -p 0.8 -a refSeq.fasta input.fastq -o output.sam

Output:
[M::mm_idx_gen::0.001*2.18] collected minimizers
[M::mm_idx_gen::0.001*2.42] sorted minimizers
[M::main::0.001*2.41] loaded/built the index for 1 target sequence(s)
[M::mm_mapopt_update::0.001*2.34] mid_occ = 10
[M::mm_idx_stat] kmer size: 19; skip: 10; is_hpc: 1; #seq: 1
[M::mm_idx_stat::0.001*2.29] distinct minimizers: 1446 (100.00% are singletons); average occurrences: 1.000; average spacing: 7.474; total length: 10808
[M::worker_pipeline::11.214*2.91] mapped 45444 sequences
[M::main] Version: 2.24-r1122
[M::main] CMD: /home/minion/.geneious_plugins/Minimap2/minimap2_linux -x map-pb --frag=yes –

secondary=yes -N 5 -p 0.8 -a -o output.sam refSeq.fasta input.fastq
[M::main] Real time: 11.215 sec; CPU: 32.579 sec; Peak RSS: 23.373 GB

✔ **Assembled**

Contig - 39,932 Reads assembled to WSLV_SA999

Attachment

RefSeq.gb

Consensus.fasta:

Alignment of consensus and Ref.seq.fasta
