# Supplementary material for: Mosquito-independent milk-associated transmission of zoonotic Wesselsbron virus in sheep
Source: PLoS Pathog. 2024 Dec 9;20(12):e1012751. doi: 10.1371/journal.ppat.1012751 (PMC11658706; doi:10.1371/journal.ppat.1012751)
Supplement: S2 Appendix — (PDF) [file ppat.1012751.s005.pdf]

S2 Appendix. Sequencing Report – WSLV SAH177

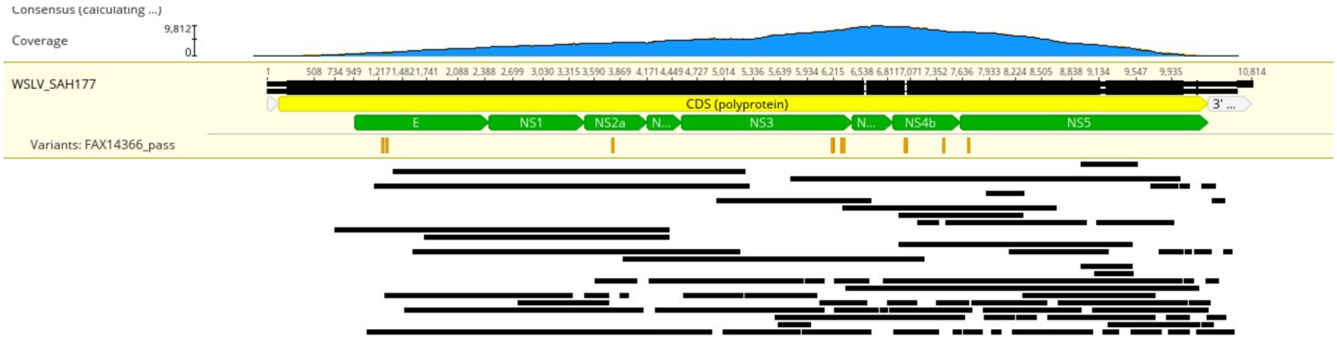

***Figure 1: Coverage and depth graph**. FAX14366bc57 43404 reads were assembled to sequence RefSeq WSLV_SA177*

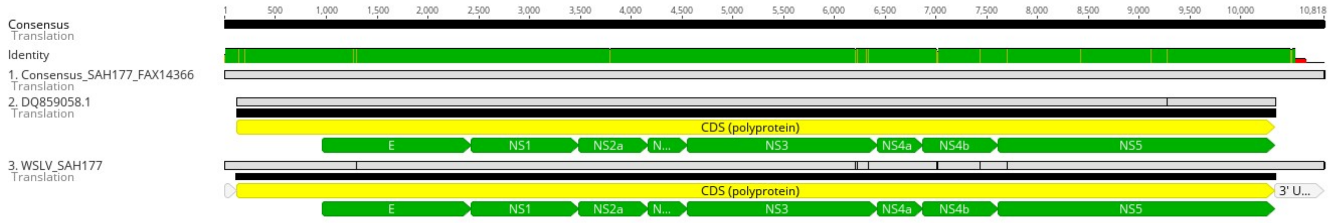

***Figure 2: Alignment graph.** It shows an alignment of the Consensus_SAH177_FAX14366bc57, Ref Seq WSLV_SA177 and DQ859058.1. Disagreements are highlighted in black.*

Consensus

Threshold 65%, if there was no coverage, it called ?, if there was lower depth as 10, N was called

Nucleotide Statistics:

Depth Mean: 5370.3 Minimum: 0 Maximum: 9812
Coverage: Ref-Seq: 98.3% (10,631 of 10,814)
5prime End: no coverage 3; depth below 10: 2
3prime End: no coverage 10639-10814; depth below 10: 10533-10638

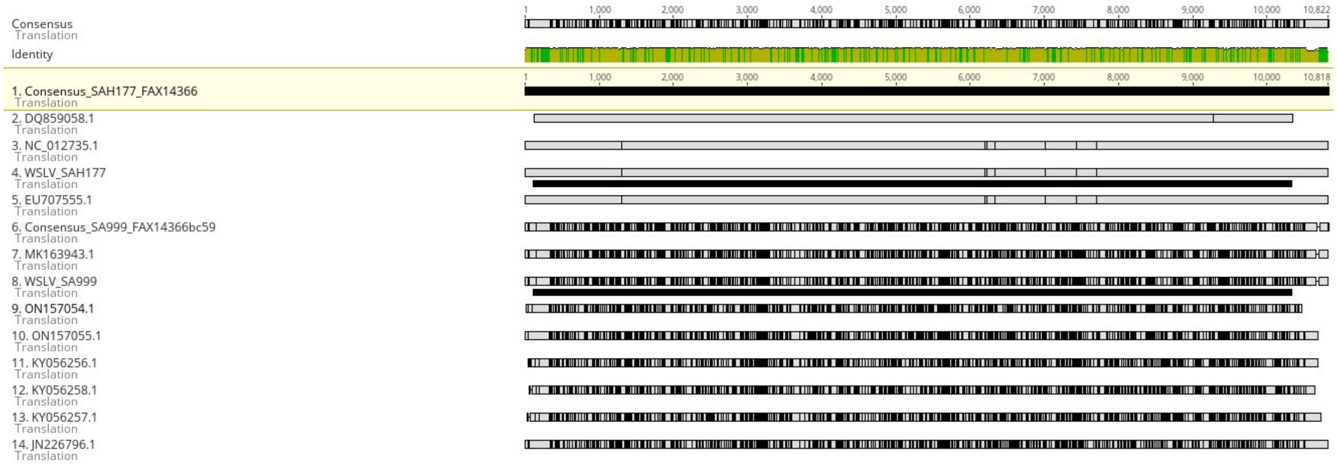

***Figure 3: MAFT alignment of all published complete WSLV genomes** -MAFT alignment NCBI WSLV.fasta*

Variant Table 1– reads mapped to DQ859058.1

Name	Minimum	Maximum	Change	Polymorphism Type	Coverage	Variant Frequency	Amino Acid Change	CDS	Codon Change	Protein Effect
A	1150	1150	G -> A	SNP (transition)	1210	30.7%	G -> S	CDS (polyprotein)	GGT -> AGT	Substitution
T	6194	6194	C -> T	SNP (transition)	8219	29.8%	A -> V	CDS (polyprotein)	GCC -> GTC	Substitution
C	9151	9151	G -> C	SNP (transversion)	3132	81.3%	E -> Q	CDS (polyprotein)	GAA -> CAA	Substitution

Sequence variants of the SAH177 virus working stock compared to the supplier’s (EVAg) provide sequence (Genbank DQ859058.1) [1].

Variant Table 2– reads mapped to RefSeq SAH177 = NC_012735.1 = EU707555 = reference strain of NCBI Virus

Name	Minimum	Maximum	Change	Polymorphism Type	Coverage	Variant Frequency	Amino Acid Change	CDS	Codon Change	Protein Effect
A	1268	1268	G -> A	SNP (transition)	1215	30.6%	G -> S	CDS (polyprotein)	GGT -> AGT	Substitution
T	1306	1306	C -> T	SNP (transition)	1293	82.6%		CDS (polyprotein)	TGC -> TGT	None
A	3794	3794	G -> A	SNP (transition)	4201	62.5%	G -> R	CDS (polyprotein)	GGA -> AGA	Substitution
G	6205	6205	T -> G	SNP (transversion)	8010	86.8%	F -> L	CDS (polyprotein)	TTT -> TTG	Substitution
A	6223	6223	G -> A	SNP (transition)	8076	78.8%		CDS (polyprotein)	AAG -> AAA	None
T	6312	6312	C -> T	SNP (transition)	8184	29.8%	A -> V	CDS (polyprotein)	GCC -> GTC	Substitution
G	6334	6334	A -> G	SNP (transition)	8436	75.6%		CDS (polyprotein)	AAA -> AAG	None
	7010	7010	-C	Deletion	9311	78.8%		CDS (polyprotein)		Frame Shift
C	7018	7017	(C)4 -> (C)5	Insertion (tandem repeat)	9324	46.2%		CDS (polyprotein)		Frame Shift
T	7432	7432	C -> T	SNP (transition)	8024	68.5%		CDS (polyprotein)	ACC -> ACT	None
T	7703	7703	C -> T	SNP (transition)	7673	87.8%	P -> S	CDS (polyprotein)	CCA -> TCA	Substitution

Sequence variants of the SAH177 virus working stock compared to the strain reference sequence (GenBank NC_012735.1; identical to EU707555).

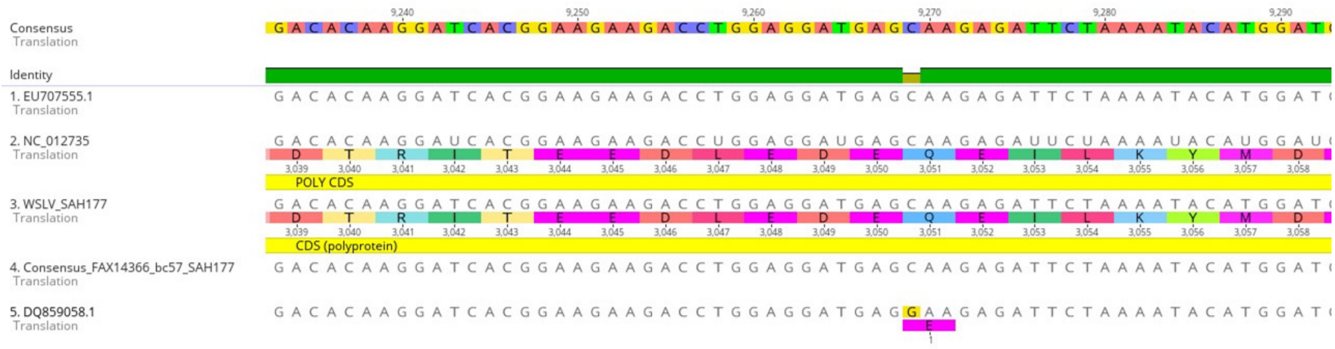

Figure 4: Amino acid (AA) 3051 (nt 9151) in NS5. *As shown in Table 2 above, the SAH177 working stock (inoculum) has a Q at the AA position 3051 (nt 9151)of the polyprotein within NS5 as in the reference sequences (RefSeq=NC_012735 / EU707555.1/WSLV_SAH177). However, DQ859058.1 has an E at this position.*

Geneious assembly report

✔ 18,844 of 34,314 reads were assembled to WSLV_SAH177 to produce Contig
✖ 15,470 reads were not assembled

Assembled 89 documents using Minimap2. Show Options
Assembly Duration: 16 minutes and 20 seconds

Note: Minimap2 produced 58,874 output reads from 34,314 input reads. This may be due to Minimap2 either mapping reads to multiple locations, or splitting long reads and mapping those split reads to different regions of the reference sequence(s).

Command Line:
minimap2_linux -x map-pb --frag=yes --secondary=yes -N 5 -p 0.8 -a refSeq.fasta input.fastq -o output.sam

Output:
[M::mm_idx_gen::0.001*2.58] collected minimizers
[M::mm_idx_gen::0.001*2.70] sorted minimizers
[M::main::0.001*2.68] loaded/built the index for 1 target sequence(s)
[M::mm_mapopt_update::0.001*2.61] mid_occ = 10
[M::mm_idx_stat] kmer size: 19; skip: 10; is_hpc: 1; #seq: 1
[M::mm_idx_stat::0.002*2.54] distinct minimizers: 1430 (100.00% are singletons); average occurrences: 1.000; average spacing: 7.562; total length: 10814
[M::worker_pipeline::12.034*2.92] mapped 34314 sequences
[M::main] Version: 2.24-r1122
[M::main] CMD: /home/minion/.geneious_plugins/Minimap2/minimap2_linux -x map-pb --frag=yes --secondary=yes -N 5 -p 0.8 -a -o output.sam refSeq.fasta input.fastq
[M::main] Real time: 12.034 sec; CPU: 35.180 sec; Peak RSS: 15.102 GB

✔ Assembled

Contig - 43,404 Reads assembled to WSLV_SAH177

Attachment

RefSeq.gb

Consensus.fasta:

Alignment of consensus and Ref.seq.fasta

2x Variant table

MAFT alignment NCBI WSLV.fasta

References

1. Grard G, Moureau G, Charrel RN, Holmes EC, Gould EA, de Lamballerie X. Genomics and evolution of Aedes-borne flaviviruses. J Gen Virol. 2010;91(Pt 1):87-94. Epub 20090909. doi: 10.1099/vir.0.014506-0. PubMed PMID: 19741066.
