# Supplementary material for: Mosquito-independent milk-associated transmission of zoonotic Wesselsbron virus in sheep
Source: PLoS Pathog. 2024 Dec 9;20(12):e1012751. doi: 10.1371/journal.ppat.1012751 (PMC11658706; doi:10.1371/journal.ppat.1012751)
Supplement: Attachment — Submitted filename: ResponseToReviewers.pdf [file ppat.1012751.s009.pdf]

Point by point replies to reviewers' comments

Part I - Summary
Please use this section to discuss strengths/weaknesses of study, novelty/significance, general execution and scholarship.

Reviewer #1: The manuscript entitled "Mosquito-free milk-associated transmission of zoonotic Wesselsbron virus in sheep", describes an interesting, primary descriptive, study in which an extensive data package is presented in a sound and conclusive way that sheds new light on mosquito-independent (vertical) transmission of WSLV, most likely mediated by milk. The unique experiment paves the way for similar studies with other arboviruses that may use this route as well and opens opportunities for more mechanistical/fundamental study approaches. This being said, the manuscript could be improved by addressing the following points.

Reviewer #2: The authors conducted a very interesting and novel study by inoculating ewes with WSLV from two different clades. Instead of pregnant ewes, this study utilized ewes who had nursing young lambs. The study provides a wealth of information about viral RNA copies in different tissues and secretions, clinical biochemistry, macroscopic pathology, histopathology and neutralizing antibodies. All of this is very valuable data for future studies. It is indeed very surprising that WSLV can be transmitted to lambs via milk. It has always been assumed that the virus crosses the placenta or that lambs become infected by nasal secretions from their dam or that transmission is via mosquito bites.
This paper also provides more evidence that Clade II viruses may be neurotropic and Clade I viruses hepatotropic. This has important implications for the diagnosis of WSL disease. Traditionally, only liver samples are tested for WSLV when a case is suspected.
The manuscript is well written and there are only minor grammatical errors.

Response: We are grateful for the positive comments and the constructive and thorough critiques by both reviewers. The requested changes have greatly improved our manuscript.

Part II – Major Issues: Key Experiments Required for Acceptance
Please use this section to detail the key new experiments or modifications of existing experiments that should be absolutely required to validate study conclusions.

Generally, there should be no more than 3 such required experiments or major modifications for a "Major Revision" recommendation. If more than 3 experiments are necessary to validate the study conclusions, then you are encouraged to recommend "Reject".

Reviewer #1: (No Response)

Reviewer #2: No major modifications required.

Response: Thank you.

Part III – Minor Issues: Editorial and Data Presentation Modifications
Please use this section for editorial suggestions as well as relatively minor modifications of existing data that would enhance clarity.

Reviewer #1:
1) Title; it might be better to state mosquito-independent instead of mosquito free.
Response: The title was adapted accordingly.

2) The short title is longer than the normal title
Response: The short title is now shorter: "Direct transmission of Wesselsbron virus to suckling lambs"

3) The ‘flow’ in the abstract is not optimal and could benefit from some editing.
Response: We have made some changes and hope to have improved the flow of the abstract.

4) Intro: Line 1: epidemics of what?
Response: We have specified “viral diseases”.

5) The age, lambing history and origin of the individual ewes is not reported, nor the randomization strategy. Please add this information. Furthermore, from the results section it is suggested that the 3 groups were housed in different stables though this is not explicitly stated.
Response: We have tabulated the missing information into the S1 Table. The results text (p.4) now reads “*…lactating ewes with their respective lambs were randomly successively allocated to the mock group, then to one and the other group to be inoculated. The groups were housed in three different BSL3Ag biocontainment stables.*”. The methods section (p. 12) is also more explicit “*Each group was housed in a different isolation BSL3Ag stable.*” Regarding randomization, please note that the method already stated that “WSLV inoculation was blinded with respect to the virus strain.”

6) The section “Virus stocks for in vivo studies” does not read well. Furthermore, it does not becomes clear whether the rescued viruses used to inoculate were confirmed by sequencing.
Response: The sequence of the virus inoculum of rSA999 and SAH177 were confirmed by sequencing showing 100% sequence identity for the recombinant virus rSA999, and a few variants in the SAH177 sequence. A section regarding the sequencing method was added to the methods (p. 12) and the details of sequence assembly and the consensus sequences are now included in S1-S4 Appendices.

7) In addition to point 6, have the authors sequenced samples (including milk samples) coming out of the trial to assess for the presence of adaptive mutations?
Response: Unfortunately, we did not collect the necessary samples from milk or from lamb tissues for virus isolation or full genome sequencing to demonstrate potential adaptive mutations.

8) The limit of detection for the PCR is set to 10 genome copies however it can be clearly seen that in the lower ranges, samples tested either 10^3 or negative. Consequently it is very unlikely that the lower limit of detection is 10. Please explain and/or adapt.
Response: Indeed, this was an annotation mistake and we are grateful for this remark. The method can detect 10 viral copies per reaction (2ul) and not 10 copies/g of tissue. We have now corrected the figure axis labels, the limit of detection line, and the corresponding figure legends accordingly. The following sentence is now included in results (p. 6): “*Variability in daily copies in some nasal and ocular conjunctival samples may in part be explained by the alternate sampling of the left and right sides.*”

9) Fig 3: the scale is log 10 though linear values are presented
Response: Thank you for spotting this versioning error, the axis label has now been updated to match the linear scale of the axis.

10) Fig 2 F: there is one sample highly positive at day 5 but related samples at day 3 and 6 seem to be negative. This seems biologically highly unlikely. Could you please explain.
Response: Figure 2F (now Fig. 2G) showed viral RNA copies in nasal swabs of ewes. To reduce the burden of the sampling procedure for the animals, the daily nasal and conjunctival swab samples were taken alternatively from the left and right sides. This is now specified in methods (p. 12). The alternate sampling site likely explains part of the daily variability in these datasets.

11) Have the authors assessed potential replication site in the udder, or how would the virus end up in the milk?
Response: The possibility of replication in the udder cannot confirmed and cannot be excluded either. The following text was added in the discussion: “*WSLV replication in the udder could not be confirmed by immunohistochemical analysis for NS1 staining in the mammary gland of any ewe on 12 dpi. While these data do not formally exclude local virus replication, additional pathways may also contribute to*

high virus titers in milk including vascular leakage in highly viremic animals, and virus transport within infected immune cells transmigrating from the blood to the milk."

12) The horizontal/vertical transmission route as presented might also impact transmission dynamics from an epidemiology view. With one mosquito multiple animals may become infection (one ewe and its lambs). Have the authors fed this data into transmission models?
Response: This is an interesting point that is beyond our expertise and could be further explored.

13) Could transmission also be the other way around. From lambs to ewes. The dataset possibly does not allow any conclusions here but this could be discussed in the discussion section.
Response: This is an interesting possibility that would have required a different experimental design. The following text has been added to the discussion (p. 9): "*Our study design did not permit to test the opposite route of transmission, from lambs to ewes. Indeed, retrograde infection via milking procedures is likely the principal transmission route in the current epidemic of H5N1 highly pathogenic avian influenza clade 2.3.4.4b in dairy cows in United States (https://doi.org/10.1038/s41586-024-08063-y).*"

Reviewer #2:

INTRODUCTION:
Remove the word 'However' from this sentence or replace with 'For instance': However, the outbreaks of Zika virus (ZIKV) in South America and West Nile virus (WNV) in North America……
Response: We have changed the text accordingly and added "*For instance, …*"

Regarding this sentence: Natural outbreaks of WSL disease are clinically similar to Rift Valley fever with abortions and death of newborn lambs, which requires proper diagnostic confirmation that is often absent in resource poor regions of Africa.
It is not that diagnostic services are absent. There is in fact fairly good diagnostic services in many African countries. It is more that lambs die from so many causes that farmers often don't attempt to have a diagnosis made. Sheep are also often farmed extensively in arid areas, and abortions or lamb deaths are not detected soon enough to make a diagnosis possible. Fetuses with malformation are sometimes presented for necropsies but the diagnosis cannot be made at this stage since viral RNA is no longer detectible in the tissues. This study also reiterates that the macroscopic lesions and histological lesions are unremarkable in most cases.
Rather something like….. which requires appropriate sampling and multiple diagnostic techniques to confirm the diagnosis. Or …..can be challenging to diagnose since lesions are unremarkable or non-specific in most cases.
Response: We regret that the sentence was poorly formulated as we did not mean to suggest that diagnostic services were absent, but rather that diagnostic confirmation was rarely done or possible. The text has been updated accordingly to say: "*which requires appropriate sampling and multiple techniques to confirm the diagnosis.*"

PAGE 4, PARAGRAPH 2: This sentence: A more recent study using the clade I strain SAH177 [27], which was isolated from a human and passaged in mouse brains and Vero cells, reported early pathogenesis of WSLV infection in pregnant ewes [28].
I believe SAH177 is a clade II strain.
Response: The typo has been corrected. Thank you.

Reference 27 also seems to in the wrong place. Passaged in mouse and Vero cells in 1957 [27], reported…..
Response: The reference was moved accordingly.

There is not much is mentioned about the pathogenicity of clade II strains in this paragraph. I suggest adding a line of 2 about this.

It seems that not much is known about the pathogenicity of SA999 (clade II) in sheep as well. However, the VanTonder strain, also in clade II, was inoculated into 30 pregnant ewes [Weiss et al] of which 4 gave birth to weak lambs that died or were killed in extremis at 2 to 3 dpi and 8 aborted between 4 and 7 dpi. Six of the ewes died at which point the experiment was terminated due to biosafety concerns.
Response: The reviewer likely meant that both SA999 and the van Tonder strain are both clade I (not II). We have added the following short section further describing the results of the seminal study of Weiss et al.: "*The pathobiology of clade I WSLV strains has not been studied since the seminal description of WSL disease by Weiss et al. (van Tonder strain) [12]. Experimental inoculation was the most severe and fatal in newborn lambs, while the disease was milder and resolved in older lambs. A large cohort of pregnant ewes were then infected intravenously with the van Tonder strain leading to birth of fatally sick animals, abortions, and death of ewes all within a week of infection, when the experiment was terminated because of biosafety concerns [12].*"

RESULTS:
PAGE 6, UNDER THE HEADING: High WSLV titers in milk of infected ewes correlates with transmission to lambs
The overlapping lines in Figure 2C is difficult to interpret. Many of the symbols overlap making it impossible to tease data for different animals apart. Split this graph into 2 graphs like the 2 graphs for the serum samples.
Response: We completely agree that Figure 2C was unclear, and it was split into two panels as requested to enhance readability.

PAGE 7, VIRAL LOAD IN ORGANS:
The description of how many organs was positive for the ewes inoculated with SAH177 is not clear. Rewrite to make the meaning clear.
This sentence: The ewe with the shortest viremia was RT-qPCR negative in the rest of tested organs, whereas the four other ewes had detectable virus in the tonsils, and in one or two additional samples including spleen, lymph nodes, kidneys, and mammary gland.
From the graph in Figure 4 it seems that 3 ewes (and not 4 as stated) had virus in the tonsils and 3 had virus in the spleen (not one or 2 as stated). It is also difficult to interpret the data in the graphs.
Response: Many thanks for these comments. Figure 4 has now been completely redrawn to show each organ in a different panel, which greatly simplifies data description, presentation, and analysis. The text and figure legend have been verified and adapted as needed to accurately reflect the data shown:
"*In ewes inoculated with SAH177, the liver was the only organ where the virus persisted in all animals at the end of the study (Figure 4I). In the other organs, the detection of viral RNA was highly variable: one ewe had detectable virus in most tissues (blue rhombus), while the ewe with the shortest viremia (blue square) was RT-qPCR negative in all other organs. Few organs were positive for virus RNA in the three other ewes (Figure 4A-K).*"

PAGE 10, FIRST PARAGRAPH: This sentence: Moreover, results suggest that the two strains may differ in their tissue tropism preferences: neurologic (SAH177) and hepatic (SA999).
This is a very important finding. Brain samples are rarely submitted for HE and IHC for WSL diagnosis whereas liver samples are routinely submitted. It seems from this study that at least one ewe and one lamb only had lesions in the CNS and not in the liver. However, it also seems that a variety of organs (including brain samples) were positive using RT-qPCR. This implies that brain samples should be routinely submitted to detect infections with clade II viruses and that RT-qPCR should also be routinely conducted on these samples since the lesions are often non-specific and IHC negative.
Response: We agree that brain samples should be more systematically submitted for histopathological analysis in suspected cases of WSL disease. However, for WSLV diagnosis purposes, RT-qPCR of the liver samples were positive for 100% of the ewes and 100% of the viremic lambs in both groups at 12 dpi. Therefore, we believe that the liver remains the best tissue for diagnostics. Regarding the brain, we performed RT-qPCR and histology in samples from several locations of the brain (including olfactory bulbs, cerebral cortex, thalamus and cerebellum). There was no clear correlation between lesions and RT-qPCR positive result in any region of the brain at 12 dpi. Therefore, based on our results, it is difficult

to provide a clear routine sampling guideline of brain samples for diagnostic purposes. A sentence was added in the results section (p. 7): "*Overall, the liver appears as the most reliable organ to sample for WSLV diagnostic purposes regardless of the age of the animals and the clade.*"

PAGE 11, FIRST PARAGRAPH: This sentence: Similarly, TBEV is secreted……
Write TBEV out since there does not seem to be any mention of this virus elsewhere in the text.
JEV as well in the next paragraph.
Rift Valley fever and yellow fever was written out elsewhere. Either write all names of viruses out or use abbreviations consistently. However, it is easier to follow text if names that are not often used are spelt out.
Response: The different virus names have been spelled out to ease reading.

Page 1, last paragraph: '….inoculated with the clade I SAH177 strain. In contrast, the clade II rSA999 strain induced a more severe…..'
That should be clade II SAH177 strain and clade I rSA999 strain.
Response: Thank you! All mentions of WSLV clades have been double-checked and corrected as needed throughout the manuscript.

PAGE 11, LAST SENTENCE:
The paper by Le Roux (1959, The histopathology of Wesselsbron disease in sheep) should be read and cited here. The histopathology of the liver in natural Wesselsbron cases can indeed be difficult to interpret (non-specific). There is no typical pattern for degenerative changes in hepatocytes which may be centrilobular, periportal or diffuse. IHC positive cases sometimes only have rare hypereosinophilic, shrunken hepatocytes with rare IHC positive hepatocytes and Kupfer cells. Other cases have diffuse necrosis that looks very similar to RVFV infection in lambs. The only consistent finding seems to be proliferation of Kupffer cells. Unfortunately, natural cases are rarely found, and the histopathology still requires more study.
Response: Thank you for the suggestion. We have now cited this reference as follows: "*Histological hepatic lesions were mostly of mild intensity and corresponded to the non-specific pattern of degenerative changes in hepatocytes previously reported [14, 26, Le Roux 1959], ...*"

PAGE 12, SECOND PARAGRAPH: This sentence: These findings are striking because there is only little evidence of encephalitis experimentally infected animals, which is has been described mostly in newborn and aborted lambs [14, 28].
Remove this sentence or modify.
Coetzer et al 1978 [your reference 14] distinctly mentions that encephalitis was not found in any of the brain specimens. Oymans et al. 2020 only mentions strong staining of WSLV antigen throughout the fetal brain that included differentiated neurons, neuroglial cells and microglial cells with the formation of microglial nodules. I think the authors of the new research can claim that encephalitis with neuronal cell death accompanied perivascular cuffing is a finding that has not been described before (unless the authors can point a paper out that I missed). This has important implications for diagnostics. A recommendation should be added that when Wesselsbron is suspected, brain samples should be collected in addition to liver samples, from multiple location and histology and RT-qCR done. Can the authors recommend which areas the brain they consider critical or most useful for sampling?
Response: The sentence has been modified to be more decisive about the two cited papers. Note that in the article by Le Roux (1959) mentioned in the point above, extensive meningo-encephalitis was actually noted in foetuses of vaccinated ewes. The new sentence is as follows:
"*These findings are striking because, to our knowledge, this is the first documented case of WSLV-associated meningoencephalitis in ewes and horizontally infected lambs [14, 28]. In previous studies, meningo-encephalitis and brain malformations were only found in aborted and stillborn lambs infected during gestation when ewes were immunized with the live attenuated Ondertespoort WSL vaccine strain, or from ewes that received combined immunization for WSL and Rift Valley fever [24, 39].*"

As mentioned above, our RT-qPCR results from the brain samples do not allow us to make a specific sampling recommendation of the brain for diagnostics.

Also, describe the origin of the SAH177 strain better. A field worker became ill with what was assumed to be encephalitis. No symptoms were described that suggests hepatic disease. Therefore, it is assumed that this virus, and by extension the Clade II viruses, are neurotropic. I also suggest that the authors mention the Onderstepoort Wesselsbron vaccine strain that causes malformations of the CNS in sheep fetuses. Also note that RVFV becomes neurotrophic when passaged in the brains of new-born mice. However, I am not sure if the original stain used to produce Smithburn vaccine was determined to be neurotropic rather than hepatotropic. This might be interesting to add.
Response: We have provided further information on the SAH177 strain in methods (p. 14) as follows: "*Clade II WSLV SAH177 99871-2 strain was provided by European virus archive global (EVAg, Marseille, France). SAH177 was originally isolated from a human subject in 1955 in South Africa [27], and passaged 21 times in suckling mice. It was donated by the University of Texas Medical Branch to the EVAg, where it was passaged at least 6 times in C6/36 cells (GenBank: DQ859058.1)[29]. The virus was passaged 3 times in C6/36 cells at the IVI to generate the working stock.*"
The text has been updated accordingly (p.10/11): "*Similarly, the attenuated Rift Valley fever Smithburn vaccine strain, generated by serial passage in mouse brain, was more neurotropic and less hepatotropic (Smithburn, 1949).*"

FIGURE 2C: Too many overlapping lines and symbols. Split into 2 separate graphs for rSA999 and SAH177.
Response: The figure has been modified accordingly.

FIGURE 4: Put the data in tables instead of graphs. It is very difficult figure out which organ was positive in each animal.
Response: As indicated above, we have now expanded the figure into multiple panels, which greatly helps readability.

FIGURE 6: Lengthen the arrow heads so that the tip points exactly to the lesion. At the moment they are triangles and one has to guess which tip of the triangle points to the lesion.
Response: The figure and the legend have been modified accordingly.
